# Vasculature deprivation – induced osteonecrosis of the rat femoral head as a model for therapeutic trials

**DOI:** 10.1186/1742-4682-2-24

**Published:** 2005-07-05

**Authors:** Jacob Bejar, Eli Peled, Jochanan H Boss

**Affiliations:** 1 Department of Pathology, The Bnai-Zion Medical Center and The Bruce Rapapport Faculty of Medicine, Technion-Israel Institute of Technology, Haifa, Israel; 2 Department of Orthopaedic Surgery B, Rambam Medical Center, and the Bruce Rappaport Faculty of Medicine, Technion-Israel Institute of Technology, Haifa, Israel

## Abstract

**Experimental Osteonecrosis:**

The authors' experience with experimentally produced femoral capital osteonecrosis in rats is reviewed: incising the periosteum at the base of the neck of the femur and cutting the ligamentum teres leads to coagulation necrosis of the epiphysis. The necrotic debris is substituted by fibrous tissue concomitantly with resorption of the dead soft and hard tissues by macrophages and osteoclasts, respectively. Progressively, the formerly necrotic epiphysis is repopulated by hematopoietic-fatty tissue, and replaced by architecturally abnormal and biomechanically weak bone. The femoral heads lose their smooth-surfaced hemispherical shape in the wake of the load transfer through the hip joint such that, together with regressive changes of the joint cartilage and inflammatory-hyperplastic changes of the articular membrane, an osteoarthritis-like disorder ensues.

**Therapeutic Choices:**

Diverse therapeutic options are studied to satisfy the different opinions concerning the significance of diverse etiological and pathogenic mechanisms: 1. Exposure to hyperbaric oxygen. 2. Exposure to hyperbaric oxygen and non-weight bearing on the operated hip. 3. Medication with enoxaparin. 4. Reduction of intraosseous hypertension, putting to use a procedure aimed at core decompression, namely drilling a channel through the femoral head. 5. Medication with vascular endothelial growth factor with a view to accelerating revascularization. 6. Medication with zoledronic acid to decrease osteoclastic productivity such that the remodeling of the femoral head is slowed.

**Glucocorticoid-related osteonecrosis **appears to be apoptosis-related, thus differing from the vessel-deprivation-induced tissue coagulation found in idiopathic osteonecrosis. The quantities of TNF-α, RANK-ligand and osteoprotegerin are raised in glucocorticoid-treated osteoblasts so that the differentiation of osteoclasts is blocked. Moreover, the osteoblasts and osteocytes of the femoral cortex mostly undergo apoptosis after a lengthy period of glucocorticoid medication.

## Background

Osteonecrosis of the femoral head is of both clinical and economic interest, nearly 20,000 patients being hospitalized annually in the U.S.A. for treatment of this disease. Different risk factors have been discussed, yet the etiology and the pathogenesis of osteonecrosis are still uncertain [1]. Clinical trials of novel therapeutic modalities are hindered by the lack of a suitable experimental model of the human disease [2]. Osteonecrosis is either "idiopathic" in nature or incidental to one of a number of diseases. To discover the chain of events resulting in osteocytic death, be it by necrosis or apoptosis, experimental models ought to replicate a "circulatory-deprivation" mishap, implicit in the practice among physicians of applying the epithet "avascular" to the disease. The epiphysis of the head of the femur is at particular risk of ischemic damage because it is undersupplied with effectual collateral circulation. Indeed, blood supply and drainage are provided by functional end-vessels. Irrespective of where the blood flow is initially disrupted, i.e. at the level of arteries, veins, capillaries or sinusoids, the circulation in the arteries is ultimately arrested [3].

Rodents are frequently used in preclinical tests of novel therapeutic modalities. So it behooves the reader to notice that interrupting the circulation in the femoral head of rats, with their lifelong persisting physeal cartilage, mimics children's Legg-Calvé-Perthes disease more than it resembles adult osteonecrosis [4]. Irrespective of the rat's age, the reduced uptake of bone-seeking isotopes at the sites of the necrotic bone implicates the disruption of the blood supply in triggering all cases of osteonecrosis [5].

## Osteonecrosis of the Femoral Head of the Rat

The effects of therapeutic interventions on the course of osteonecrosis of the femoral head may be studied using various models. The following model has been applied by the authors of this review: the blood supply and drainage of epiphysis are interrupted by cutting the ligamentum teres and incising the periosteum at the cervical base of the femoral head of 6 month-old rats. After the operation, the rats are placed in spacious cages such that their perambulation is almost unhindered. At the time of sacrifice, the femora are excised and fixed in formalin. The samples are embedded in paraffin after decalcification. Blocks are cut such that longitudinally orientated sections bisect the insertion of the ligamentum teres [6].

Necrosis of the adipose and hematopoietic cells is histologically evident as early as the 2nd postoperative day. Necrosis of the subchondral and trabecular bone first becomes overt on the 5th postoperative day. Repair begins soon afterwards with growth of viable tissue from the epiphyseal-capsular junction into the necrotic debris within the intertrabecular spaces. Residues of the eosinophilic, granular, necrotic marrow are no longer apparent after the 3rd week. Undifferentiated mesenchymal cells initially infiltrate the necrotic marrow and are later replaced by well-vascularized fibrous tissue, carrying with it macrophages, resorbing the dead soft tissues, and by osteoclasts, absorbing the necrotic bone. Beginning in the 3rd postoperative week, newly-formed intramembranous and appositional bone remodel the original osseous framework of the epiphysis. Unevenly contoured, recently formed bony beams crisscross the intertrabecular fibrous tissue, spanning between the viable osteoid seams and the dead trabeculae. Complete replacement of all the necrotic by living bone occurs at the 6-week interval or later. The marrow spaces are repopulated by hematopoietic-fatty tissue. The femoral heads collapse, flatten or are otherwise disfigured. The physeal cartilage is mostly unaffected. Fibrous tissue invades the joint cartilage wherever the continuity of the subchondral bone plate is disrupted. Chondroclasts erode the cartilaginous matrix. A fibrous pannus eventually covers the roughened and fibrillated surface cartilage. As judged by the lack of stainable chondrocytic nuclei, the articular cartilage is undergoing focal chondrolysis, resulting occasionally in delamination of a partly free-floating cartilaginous membrane. The tissue in the expanded joint capsule is contiguous with the pannus and fibrous tissue in the marrow spaces. A shortcoming of this model is the widespread necrosis of the rat femoral heads, sporadically extending to the articular and physeal cartilage [6].

## Disposition of the Epiphyses to Undergo Necrosis

Why is the epiphysis of the femoral head frequently affected by ischemic insults, while the diaphysis and metaphysis are spared? According to Johnson and her colleagues, the limited blood circulation accounts for the clinically high incidence of osteonecrosis of the femoral head [7]. Blood supply and drainage of the diaphysis and metaphysis depend on the nutrient, metaphyseal and periosteal arteries, which enter the bone through the foramina of the cortex. Having entered the marrow, they ramify and widely anastomose with each other. On the other hand, there is no dual supply and drainage of blood to and from the epiphysis because the femoral head is covered by cartilage. Ascending fan-like to the surface of the joint, the vessels are functionally end-arteries. It follows that the osseous-hematopoietic-fatty tissues of the epiphysis as well as the articular and the physeal cartilages are particularly susceptible to obstruction of the blood flow [8,9].

## The Fate of the Ischemia – Induced Necrotic Bone

The gradual substitution of necrotic by living bone is divided into phases, which nevertheless overlap. Oxygen- and nutrient-deprived osteocytes and marrow cells die to the nearest link with the collateral circulation. Neutrophilic infiltration characterizes the acute phase, which is rapidly followed by the chronic stage during which invasion of macrophages is dominant. Granulation tissue forms and, with time, the detritus is resorbed. The stage of repair starts with the lessening of inflammation and resorption of the dead tissues. It is set in motion by an influx of pluripotential mesenchymal cells. The environmental variations and stresses to which the cells are exposed induce the pluripotential cells to differentiate into fibroblasts, chondroblasts, osteoblasts or angioblasts. The bulk of the cells involved in the reparative process infiltrate the necrotic femoral head from the hyperplastic subsynovial layer. Repair is associated with an ingress of capillary buds, which are recruited by vascular endothelial growth factor (VEGF) and diverse cytokines, which are abundantly synthesized by and released from the synovial fibroblasts residing within the hyperplastic subsynovial cell population [10,11].

The cues that monitor the behavior of the mesenchymal cells are probably derived from the microenvironment. To exemplify, cartilage and bone are produced in areas of low and high oxygen tension, respectively. Afterwards, the cartilage is transformed by endochondral ossification into bone [12]. Eventually, biomechanically redundant bone is resorbed during the remodeling stage and the newly deposited bony trabeculae are positioned along the lines of stress, as first postulated by Wolff in 1892, in so far as the skeletal architecture is adapted to biomechanical demands [13]. Concomitantly with the osteoclastic resorption of nonessential and poorly placed osseous beams, osteogenesis of trabeculae that fit the lines of force takes place. The tissue module regulating these events is the bone multicellular unit (BMU). The BMU is made up of an intraosseous, dissecting bulge of fibrous tissue with osteoclasts positioned at its closed side and osteoblasts situated along both bony surfaces. The remodeling compartment of the BMU at the fibrous tissue-bone interface is covered by flat cells facing the marrow, and by refashioning cells, i.e. osteoblasts, on its osseous side. The outspread marrow lining cells are continuous with the osteoblasts at the fringes of the remodeling compartment. The BMU's initial activity in remodeling of the cancellous bone is the digestion of the non-mineralized matrix. Given that the natural lifespan of both osteoclasts and osteoblasts is shorter than that of the BMUs, both these cell types have to be constantly replenished for remodeling to continue. The bone lining cells replace the marrow lining cells at the termination of each episode of osteogenesis such that the BMUs are sealed. The end product of BMU activity is a bone that differs from its original counterpart in that its modification is optimally adapted to perform the biomechanical functions demanded of it [14,15].

The above-described repair of the necrotic epiphyses might suggest that the healing process restores the femoral heads to their former selves. Yet unless the necrotic segment is small or restricted to the non-load bearing part of the femoral head, this is not the case in man. The clinical condition of untreated patients goes gradually downhill. Inasmuch as the reparative properties of healthy bone are excellent, this apparent discrepancy remains to be elucidated.

## Fate of the Necrotic Femoral Head in Rats

Sevitt pioneered the prevailing explanation of avascular necrosis of the femoral head in the wake of a fractured femoral neck and the ensuing revascularization of the epiphysis [16]. In the context of the vascular-deprivation-induced model of osteonecrosis [6], analysis of the derivation of the tissues spreading into the necrotic marrow is of note. Invasion of fibrous tissue into the detritus proceeds from the hyperplastic tissue around the head and neck, remnants of living tissue, residues of the ligamentum teres, and the metaphysis (given that the physis has been breached). In view of the lifelong persistence of the rat physis, the necrotic epiphyses are mainly repopulated by tissue emanating from the expanded subsynovial compartment.

With the ingrowth of blood vessels, the reparative processes are launched by permeation of circulation-born monocytes throughout the necrotic debris. The emigrating monocytes proliferate and, having differentiated into macrophages and osteoclasts, resorb the dead tissues. Perivascular progenitor cells transform into osteoblasts, which deposit bone. In addition, the invading tissues are replete with undifferentiated mesenchymal cells, which stay dormant awaiting appropriate signals, upon which they are induced to proliferate and differentiate into fibroblasts, chondroblasts, osteoblasts, angioblasts and lipoblasts. This cascade of events, however, does not hold true for all species; thus, the undifferentiated cells in canine experimental osteonecrosis migrate first and foremost from adjacent living bone [17].

As stated above, recently deposited and mineralized bony matrix is biomechanically inferior to mature bone in respect of stiffness, strength, and toughness. Hence, the recently remodeled femoral heads do not withstand the transarticular stresses of daily activity-related loads without caving [18-20]. As rats' femoral heads always undergo total necrosis, this leads to a rapidly evolving osteoarthritis-like disorder [21]. Similarly, the revascularization-related restitution of the epiphyses by newly synthesized weak osseous trabeculae is blamed for the collapse of the femoral heads within only 4 to 6 weeks of disrupting the venous drainage of the femoral neck in minipigs [22].

**Figure 1:**
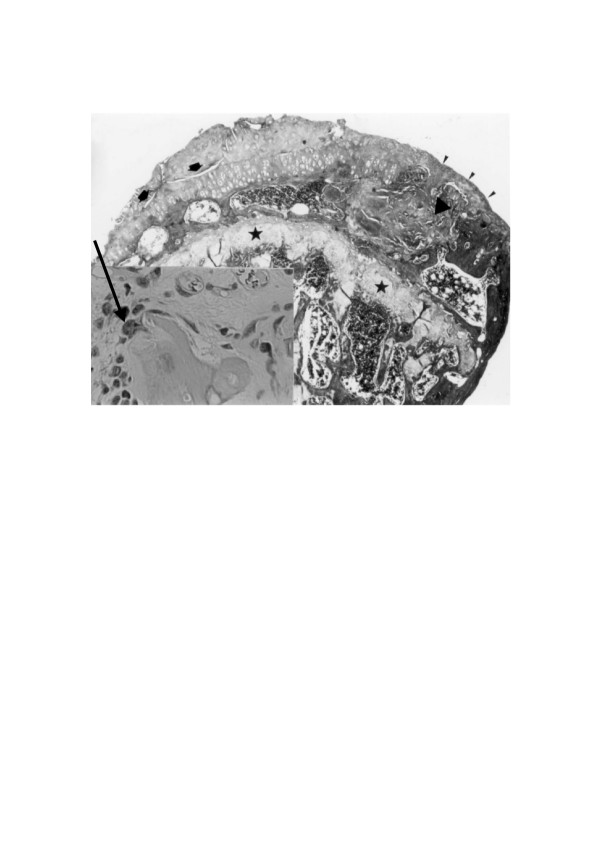
**Several fissures (arrows) split the degenerated joint cartilage**. The articular aspect of the femoral head is segmentally polished and eburnated (arrowheads). The intertrabecular spaces contain hematopoietic-fatty tissue (square) or hyalinized fibrous tissue (triangle). The physis is uninterrupted all along its path (asterisks). Inset: Residual necrotic bone within the fibrous tissue surrounded by some osteoblasts and an osteoclast (arrow).

**Figure 2:**
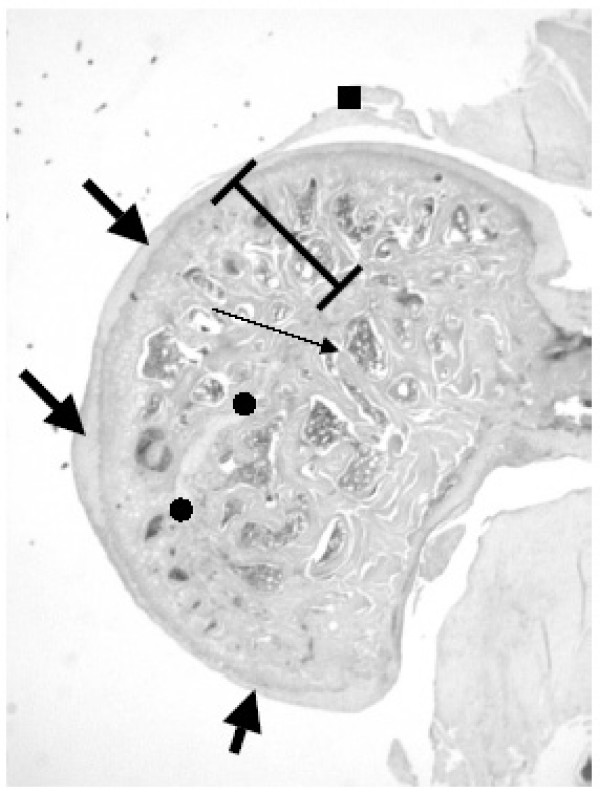
**Femoral head with AVN treated with alendronate after 42 days**. The right operated femoral head of an alendronate-treated rat. There are just remnants of the physeal cartilage (●). The physis has been breached and epiphyseal-metaphyseal bridges (long thin arrow) join the epiphyseal and metaphyseal bony trabeculae with one another. The articular cartilage is of unequal thickness (thick arrow). Even so the hemispherical configuration is preserved. The height of epiphysis is within the standard range. Remnants of the ligamentum teres (■).

The restorative activities begin during the 2nd postoperative week in the rat model. The near-hemispherical, smooth-contoured profile of the healthy femoral head is lost by the 2nd to the 3rd postoperative month. The femoral heads deviate structurally from normal shape in that they acquire a crescent-profiled, triangular or other aberrant form with rugged, murky brown joint cartilage. Not infrequently, there are no residues of dead tissues at this point in time. The amounts of newly deposited bone vary from one segment of the epiphysis to another and from one rat to another. Variously shaped and sized, lamellar-fibred or woven-fibred, newly formed trabeculae of bone crisscross the loosely to densely textured fibrous tissue that has permeated the intertrabecular spaces (Fig. 1). Cuboidal osteoblasts, often arrayed in multiple layers, abut on the lamellar-fibred or woven-fibred bone. Pseudocysts are sparsely scattered in the subchondral zone. The physis is focally or totally absent in a few cases such that the bony trabeculae of the epiphysis and metaphysis connect with one another by way of transphyseal bridges (Fig. 2). It seems that the physis is first broken up, then fibrous tissue and lastly bony beams replace the dead cartilage [9].

The articular aspect demonstrates a spectrum of changes ranging from a reduced content of glycosaminoglycan in the cartilage to a segmentally burnished and eburnated bony surface devoid of cartilage (Figs. 1 and 2). The degenerated cartilage is usually covered by a vascularized or avascular fibrous pannus. By and large, the scene at or about the 3rd postoperative month is that of osteoarthritis portraying distorted anatomical landmarks due to inappropriate repair of the epiphyseal hard and soft tissues and articular cartilage [19], matching Sokoloff's concept of degenerative joint disease as a deranged tricompartmental articulation [23].

Dead bone retains its rigid qualities for quite a long time unless it is substituted by newly formed osseous tissues. Non-remodeled necrotic bone should theoretically retain its properties of resistance to load-bearing and bending strains. There is consequently no biomechanical basis for evolving alterations of the conformation of the necrotic femoral head in the immediate period after an ischemic injury. In fact, descriptions in the literature of both functional and morphological deviations from the norm of the postosteonecrotic rat femoral head pertain to the late stage of the disease.

Human analyzers adequately and competently interpret what they perceive, but they experience difficulties in quantifying what they observe [24]. This knowledge is crucial in view of the widely-accepted supposition that the rat femoral head flattens during the early post-necrotic stage. Histomorphometrically, the height-to-width ratios and the values of the shape factor of femoral heads in rats killed 18 days after an ischemic insult differ statistically from those of rats sacrificed at an earlier time. These quantitatively-gauged statistics of remodeled femoral heads refute other authors' notions with respect to the purportedly consistent flattening, or collapse, of rat femoral heads. As a matter of fact, postnecrotic femoral heads evidently transmute into any of a number of forms during the repair stage, including femoral heads that are higher than those of healthy rats [25].

The distortion of an infarcted femoral head depends on the extent of structural degradation of its cancellous bone [26]. Because the repair processes are set in motion during the 2nd post-operative week, there is apparently no deterioration in the biomechanical properties of the femoral heads at the early stages. The differences in yield and maximum stress between the necrotic and adjacent vital bone are insignificant at the pre-deformation stage. Both parameters begin to decline with the initiation of osteoneogenesis such that they are, at this time, lower in the dead than in the contiguous living bone. The maximum stress of the adapted-sclerotic bone is higher than that of the subjacent uninvolved bone, explaining the aspherical distortion and secondary osteoarthritis of the hip at late stages of the disease [27-29].

The maximal deficit in material properties manifests itself during the mid- to late-stages of the repair phase [30], which in rats occurs a fortnight or so after the ischemic episode. Healing of the rats' injured tissues is speedy in comparison with the prolonged repair in large animal species [6]. In agreement with this paradigm, the height-to-width ratios of the femoral heads of rats killed on the 18th postoperative day clearly deviate from those of non-operated rats. Nevertheless, the direction of the shift in height-to-width ratios is unpredictable. Ratios greater than 0.4 are not encountered in non-operated rats. In contrast, height-to-width ratios greater than 0.4 are often detected on the 18th postoperative day, values ranking as high as 0.9 being occasionally encountered. There is no equivalent information in the Medline database with which these structural changes in remodeling femoral heads of rats could be compared.

Interestingly, about one third of the femoral heads of children with Perthes disease round up [31]. The epiphyseal index assigns a rank to the height-to-width ratios of femoral head contours measured by magnetic resonance imaging. Indices within the normal range are measured in children with stage I Perthes disease. These indices decrease in patients with stage II and III disease. The loss of sphericity and congruence of the femoral heads and acetabula in children with stage II and III disease coincides with flattening and widening of the epiphyses as well as with an increase in femoral head size [32]. On theoretical grounds, some authors have challenged the cascade of events mentioned above. They have postulated that the distortion of the architecture of the remodeled femoral heads in Perthes disease is secondary to the combined effects of collapse, asymmetric growth and disturbed endochondral ossification [33].

Contrary to the universally accepted paradigm, the mode by which the rats' vessel-deprived necrotic femoral heads remodel is unanticipated. The height-to-width ratios of numerous epiphyses obtained 18 and 36 days postoperatively are in fact greater than those of the control epiphyses. The adaptive reshaping of osseous tissues is responsive to alterations in the distribution and magnitude of the strain generated within the bone [34]. Comprising immature and malleable bone at the early stages after necrosis, it is hypothesized that the rat femoral heads are forced into atypical shapes by protruding from the acetabulum, or by other as yet unidentified mechanisms, such that they expand in the longitudinal direction.

Curetting the core of the necrotic epiphysis (thus stimulating osteoneogenesis) is assumed to prevent the collapse of the joint surface following blending with the subchondral bone plate of a cancellous bone-augmented vascularized fibular graft [35]. Likewise, buttressing of the remodeled epiphyses by the recently formed thick osseous trabeculae may reinforce the joint surface prior to the load-induced cave-in of the femoral heads. This mechanism possibly accounts for the protruding, rather than flattening, of the uppermost faces of the femoral head. The observation by Carter his coworkers that the perimeter of the tarsocrural joints in methylprednisolone-treated and exercised horses with a full-thickness osteochondral lesion increases within a few weeks of the operation [36] is crucial in the context of the hypothesis that post-necrotic repair processes may enlarge the articular structures. Notwithstanding the rather few and widely spaced trabeculae making up the osseous framework of remodeled rat femoral heads, these broad trabeculae (Fig. 2) seem biomechanically to equal the augmented bone volume fraction of osteoarthritic joints [37].

It is currently conjectured that, firstly, vascular impediment and defective repair capacity act in concert in causing non-traumatic variants of osteonecrosis and, secondly, the replicative potential of the osteoblasts is reduced in the living parts of the femoral head, supporting the pathogenetic role in osteonecrosis of malfunctioning of the bone-forming cells [38,39].

## Therapeutic Trials

### 1. Reduction of Intraosseous Pressure

Taking for granted the accuracy of the paradigm of the pathogenetic role of vascular deprivation and anoxia in bringing about necrosis of the femoral head, revascularization and oxygenation ought to be the paramount therapeutic modalities. As a matter of fact, both core decompression and implantation of a vascularized bone graft have met with success in rescuing patients' necrotic femoral heads. This success is attributed, at least partly, to the encouragement of ingrowth of well-vascularized fibrous tissue into the necrotic bone. The size of the necrotic zone dictates the fate of necrotic femoral heads. In rats, resorption of the epiphysis takes place at all times because their femoral heads undergo total necrosis because the blood inflow and outflow at the cervical level and ligamentum teres are completely severed [40,41]. Core decompression is assumed to decrease the intraosseous hypertension that causes destruction. True, core decompression provides relief of pain for the patient, but in the long run its effectiveness in preventing the progressive distortion of the epiphysis is, at best, debatable [42].

### 2. Intraosseous Conduit as a Model of Core Decompression

In the experience of Simank et al., drilling a sheep's epiphysis (their model of core decompression) encouraged healing of the necrotic femoral heads [43]. The authors of the present review used a rat model to study the fate of the necrotic epiphysis after creating an intraosseous conduit through the femoral head. After incising the periosteum at the cervical base and cutting the ligamentum teres, a 21-gauge needle was lanced into the foveola via the residue of the ligament and pushed in the direction of the neck up to the opposite cortical bone. Hypercellular fibrous tissue with crowding sinusoidal blood vessels replaced the hematopoietic-adipose marrow 4 to 6 weeks after the operation. Clustered osteoblasts blended with undifferentiated mesenchymal cells. Osteoclasts abutted on to the necrotic trabecular and subchondral bone. Osteoclast-type cells were also scattered in the fibrous tissue, and when mingled with the mononuclear cell infiltrate, presented a giant cell granuloma-like appearance. Excessive osteogenesis resulted in the formation of compacta-like features and epiphyseal-metaphyseal bony bridges. Fibrous tissue occasionally extended upwards, replacing the joint cartilage, or downwards into the metaphysis. Dents, deeply permeating tunnels and large circular or polycyclic cavities at the surface of the femoral heads were found by analysis of serial sections to consist of cuts through the drilling channels. The joint cartilage showed severe degenerative changes. It is noteworthy that the disfigurement of the epiphyses was more prominent in this than in the authors' other models of attempts at therapy. The myriad sinusoidal vessels and their proximity to one another indicate that the intraosseous conduits support an exaggerated revascularization of the formerly avascular femoral heads. To conclude, the above alterations are unmistakably exclusive to the healing phase of osteonecrosis of the femoral head in the presence of an intraosseous conduit [6,44].

Lancing the epiphysis with a 21-gauge needle is not expected to weaken the bone. An explanation for the conduit-related intensification of remodeling should, therefore, be sought elsewhere. 1. Conceding that a conduit accelerates the healing process as a result of its tension-lowering effect and opening up a path for vascular ingrowth, the rapid replacement of dead by living bone leads to the deposition of a weak osseous structure that is unlikely to carry weight-bearing loads without collapsing. 2. The conduit hastens the development of osteoarthritis since the osteochondral junction is inadequately reconstructed. 3. The insertion of a needle through the foveola into the epiphysis creates an inlet that permits the synovial fluid to spill from the joint cavity into the intertrabecular spaces, thus delaying the repair of bone defects. 4. The synovial fibroblasts in the distended joint capsule of rats with vessel-deprived osteonecrosis of the femoral head are jam-packed with vascular endothelial growth factor. The overexpression of this and other intermediates probably accounts for the enhanced ingrowth of blood vessels after the creation of an intraosseous conduit in the necrotic femoral heads [11,45,56].

### 3. Heparin and Low Molecular Weight Heparin

The expectation that anticoagulation would thwart osteonecrosis of the femoral head goes back to the early 1970s, when Fahlström et al. established that the incidence of osteonecrosis complicating fractures of the femoral neck was reduced nearly fourfold in patients on a daily heparin regimen as compared to a control group of untreated patients [47]. Study of the impact of heparins on revascularization and stromal cells is germane in view of the current vogue for anticoagulation of patients with osteonecrosis. In contrast to untreated rats with vessel-deprived necrotic femoral heads, nearly all the necrotic bone is resorbed in less than a month in animals receiving a daily intramuscular injection of enoxaparin at a dose of 1 mg/kg. The differences between enoxaparin-treated and untreated rats in quantities of necrotic and newly formed bone, extent of remodeling and degeneration of the articular cartilage during the repair stage are statistically significant. Indeed, slowing of the progression towards an osteoarthritis-like phenotype is a major effect of enoxaparin therapy. In vitro, heparin makes the mitogenic effect of fibroblast growth factors on endothelial cells more efficacious, stabilizes as well as protects these factors from inactivation, acts as a receptor segregating basic fibroblast growth factor, and promotes the interaction with high affinity signaling receptors on the cell surfaces. VEGF and basic fibroblast growth factor support the spread of the vasculature. These factors, which are preferentially attracted to the heparin, increase the proliferation and migration of cells associated with neovascularization. In as much as enoxaparin suppresses the reactive leukocytic response, it favors bone healing because osteogenesis is inhibited by inflammation [48-56].

### 4. Hyperoxygenation

A series of hyperbaric oxygen-treated patients with osteonecrosis of the femoral head was reported in 1990 at the 10th International Congress of Hyperbaric Medicine [57]. However, the first publication in a peer-reviewed journal about the therapeutic effects of hyperbaric oxygen (HBO) on patients with avascular osteonecrosis of the femoral head appeared belatedly 13 years later [58].

Daily exposure of patients with Steinberg stage-I osteonecrosis to HBO for 100 days reportedly results in the return to a normal MRI pattern in ~80% of cases. This cure rate compares favorably with a ~80% rate of collapse of the femoral heads in untreated patients within 4 years of the onset of the disease [59]. Yet therapeutic investigations show that hyperoxygenation has few beneficial effects on rats with necrosis of the femoral heads. This may be explained by the toxic effects of HBO or an unbalanced stimulation of cells from different lineages when a very high dose of O_2 _is employed. The in vitro upregulation of osteoclastic activity may be related to the extended exposure to O_2 _radicals. In vivo, sustained hyperoxygenation results in the production of a repair tissue replete with structurally weak collagen fibers [60-62]. Too long or too frequent exposure to HBO impacts negatively on both the structure and the mechanical properties of the bone. For instance, extensive osteolysis of living and dead bone ensues in the femoral heads of rabbits after 2 daily sessions of one hour at 2 atmospheres absolute (ATA) followed by one daily session of 3 hours at 1 ATA for 16 days and finally 2 daily sessions of 3 hours for a further 12 days at 1 ATA [63]. The breaking strength of rat bones decreases when daily exposures to HBO are extended from 4 to 6 hours [64]. Ingrowth of vessels into metaphyseal cortical defects in rats is accelerated after one daily HBO session, but is retarded when two sessions are allotted [65]. To sum up, optimal healing of a bony lesion is achieved only if exposure to HBO is restricted within an auspicious dose range.

Daily 90 minute exposures to HBO in a monoplace hyperbaric chamber enhances osteogenesis in rats after ischemic damage of the femoral heads. Hyperoxygenation is intended to uphold the innate re-establishment of well-being, and to enhance fibrogenesis, appositional and intramembranous osteogenesis, resorption of necrotic soft tissues and osteoclastic osteolysis during the late phase of osteonecrosis [66,67].

Histomorphometric parameters indicate that exposure to HBO modifies the architectural distortion of the femoral heads [63]. The HBO-mediated intensification of fibrogenesis and angiogenesis prepare the ground for the restoration of the osseous framework in the necrotic femoral heads. Unfortunately, the betterment of healing comes at the expense of an architectural disarray of the healing epiphyses with biomechanically weak bone being produced after "too great amounts" of necrotic bone are "too rapidly" replaced by immature and weak bone, so that the femoral head undergoes structural disfigurement on weight-bearing [64-67].

Exposure to HBO provides an optimal environment for repair processes as the additional oxygen carried by the circulation to ischemic sites raises the oxygen tension in the tissues. The hyperoxygenation-mediated relief of ischemia boosts the activities of fibroblasts, osteoblasts and osteoclasts in addition to supplying the extra oxygen that is indispensable for meeting the increased metabolic demands of regenerating tissues. Given that vascularization of the ischemic site is sufficient, exposure to HBO within the first 4 to 6 hours after injury achieves the optimum results [68-70]. Shifting the homeostatic environment by affecting the functions of the bone cells and mineralization of the osteoid, exposure to HBO reduces the healing time of bone fractures and beneficially influences, among other factors, the healing of non-unions. In rats, intermittent exposure to HBO hastens callus formation in fractured bones [71,72]. Treatment of spontaneous hypertensive rats with HBO averts osteonecrosis of the femoral heads [73].

The prognosis after conservative therapy of femoral capital osteonecrosis is mostly poor, osteoarthritis more often than not evolving within 2 to 3 years of the diagnosis [74]. A perfect therapeutic modality would boost the substitution of new bone in the necrotic femoral head at a pace at least as rapid as the resorption of the dead bone, such that loss of structural integrity and biomechanical adequacy would not be below the capacity of the femoral head-acetabulum couple for functionally effective load-carrying without collapse of the epiphysis [75,76]. Diverse therapeutic options are proposed to achieve this goal; for instance, bone grafting, implantation of a vascularized bone graft, core decompression, electrical stimulation and hyperbaric oxygenation.

### 5. Hyperoxygenation and Non-Weight Bearing

It is now close to half a century since HBO was first acclaimed as a beneficial adjunct to conventional therapy for miscellaneous illnesses [77-80]. An interesting proposition is to combine non-weight bearing (NWB) on the necrotic femoral head with exposure to HBO [77]. The rationale is founded on the reduction of bone marrow edema and lessening of intramedullary ischemia by elevating the arterial oxygen tension by exposing the patients with osteonecrosis to HBO. Both ischemia and edema of the marrow are critical factors in the survival of bony tissues confined by non-yielding boundaries, to wit, the rigid cortex. Ischemia and edema bring about metabolic conditions that counteract an effective osteolysis of the dead bone on the one hand and osteogenesis on the other [78,79]. Exposure to HBO enhances angiogenesis, maturation of collagen and proliferation of fibroblasts, osteoblasts and osteoclasts, all of which contribute to the speedy repair of bone lesions [80,81]. While the advantages of exposing a damaged bone to HBO are well founded, the clinical implementation of NWB as a monotherapy does not prevent collapse of the necrotic femoral head [82,83].

In a study of the combined effect of exposure to HBO and NWB on the repair of necrotic femoral heads, rats were housed in an enclosed 2 × 2 × 1.3 feet Plexiglas space, in which the hind limbs were suspended by tail traction so that the hip joints were not loaded. The trailing end of a Velcro strip, wrapped around the rats' tails, was fixated to a crossbar with a wheel and swivel assembly riding on opposite walls of the cage. Thus, the rats had freedom of movement in the longitudinal and orthogonal directions and access to food and water at all times. From the 5th postoperative day, the rats were exposed to 100% oxygen at 2.5 ATA over 22 or 32 sessions, each lasting 90 min. Control animals were treated only by NWB. The rats were killed 30 or 42 days postoperatively. There were no changes in the femoral heads of sham operated (control) rats that had been subjected to NWB, HBO, or both. The gamut of post-osteonecrotic repair activities was enhanced in rats on the HBO plus NWB regimen: osteogenesis, florid osteoblastic rimming, preosteoblasts abutting on necrotic or lately deposited bone, clustered undifferentiated mesenchymal cells in hypercellular fibrous tissue, osteoclastic osteolysis of viable and necrotic bone, chondroclastic chondrolysis and degeneration of the joint cartilage were significantly more advanced than in other reported models of therapy. Severe distortion of the femoral heads ensued in almost a third of the rats. The structural deformations manifested various configurations affecting the shape, symmetry, organization of the hard and soft tissues, and the height as well as the width of the epiphysis. The irregularly shaped femoral heads had jagged surfaces subsequent to asymmetrical resorption of the necrotic bone and erratic substitution by thriving, recently formed bone. In place of the innate, smoothly surfaced hemispherical outline of the femoral head, any of a myriad geometric configurations evolved. Loss of tissue led to localized surface depressions, which were lined by a layer of synovial-like cells several cells thick. In other instances, exuberant tissue proliferation resulted in an elevation of the articular aspect. The sporadically decreased epiphyseal height signified flattening of the bony compartments of the femoral heads. Even though remodeling and distortion often coincided, the hemispherical profile of the femoral head was every so often preserved. Where sizable parts of the epiphysis had been replaced, the cartilage, the bone, the fibrous tissue, or all of these were always accompanied by peculiar architectural modifications. The semiquantitatively gauged parameters indicating deformation were statistically less significant on the 30th postoperative day in rats treated by the combined NWB plus HBO regimen than in the rats treated with either NWB or HBO alone [6-8,12,19,22,26,44,59,77]. Yet the management of patients with osteonecrosis of the femoral head or Perthes disease by NWB is at best debatable in so far as improvement of the functionality of the hip joint is concerned [2,84-87].

### 6. Advantages and Disadvantages of Hyperoxygenation

Several studies have established the favorable effects of HBO therapy on the course of certain ischemia-induced conditions, but there is no consensus about its therapeutic value in osteonecrosis of the femoral head [88].

Vessel-deprived epiphyseal osteonecrosis in rats does not fully imitate all the clinical, humoral and metabolic conditions that precede the disease in man. Nevertheless, the causal pathway of impeded blood supply and drainage is embodied in most experimental models of the disorder [6,89]. The versatile HBO therapy opposes the progression of necrosis and expedites reparative processes. Theoretically, the fibrous tissue enclosing the bone acts as a barrier that prevents oxygenation of the vessel-deprived region [90]. Practically, this barrier is overcome by the large amount of serum-dissolved O_2 _which, after HBO medication, increases the diffusion distance notwithstanding the fibrous tissue enclosure. The hyperoxygenation-induced relief of marrow edema is a spin-off of HBO exposure; it is the byproduct of reflex vasoconstriction and oxygen-induced osmosis, which reverses the usual pumping mode of interstitial fluids, i.e. from the tissues back into the circulation. Hyperoxygenation also induces the precursors of the multipotential mesenchymal cells to mature into osteoblasts and at the same time encourages osteoclastic osteolysis such that remodeling is enhanced overall [90-96]. Finally, HBO-induced suppression of the inflammatory response promotes osteogenesis [97]. Acting in concert, these consequences of HBO therapy influence the cascade of events so that bone turnover is accelerated. Alas, all the advantages gained by HBO exposure come at a price. True, hyperoxygenation results in rapid removal of the necrotic debris and a speedy rebuilding of a viable bone; but having been just lately deposited and mineralized, this bone is biomechanically weak. In fact, daily ambulation suffices to distort the architecture of the femoral head, and the evolution of an osteoarthritis-like disorder is just a matter of time [29].

### 7. Medication with Vascular Endothelial Growth Factor

VEGF stimulates angiogenesis, recruitment and migration of osteoblasts and activation of osteoclasts. So far so good; but medication with VEGF would also enhance the removal of dead bone and increase the formation of a mechanically weak intramembranous bone, two events that ought to be avoided at all costs. In the context of fracture healing, a slow VEGF-releasing device is an effective therapeutic mode [98-102], but its efficacy in the treatment of femoral capital osteonecrosis is doubtful, considering that the para-articular apparatus is already jam-packed with VEGF-containing synovial fibroblasts [11]. Contrary to the widely accepted goal of supporting angiogenesis, the authors of this review are convinced that release of VEGF should be inhibited [103]. Given that the ingrowth of blood vessels into the necrotic epiphysis sets in motion a cascade of events terminating in the destruction of the femoral head, whether partial or total, arresting the release or activity of VEGF may possibly slow down the rapid impairment of the biomechanical properties of healing bone. Åstrand and Aspenberg have arrived at a similar conclusion, albeit in a different model. During the ingrowth of osseous tissues into a bone graft placed in a bone chamber, the necrotic debris was not resorbed in rats treated with alendronate but was more or less removed in their untreated counterparts [104]. By analogy, the structural failure of necrotic femoral heads in patients begins with the resorption of dead bone during the revascularization phase prior to the point in time at which sufficient new osseous matrix has been synthesized and mineralized, i.e. that the skeleton has been adequately reinforced. Otherwise, daily load-bearing of the hip would deform the femoral head. If the early resorption of necrotic subchondral and trabecular bone could be minimized, premature structural breakdown of the femoral head should be averted and the ensuing osteoarthritis may be prevented or at least slowed down [21,105].

Lieberman et al. recommended combining core decompression with VEGF medication so as to strengthen "patients' angiogenic potential" [106]. This proposal is diametrically opposed to the concepts of the authors of this review. Firstly, the cells of the hyperplastic para-articular apparatus of rats with osteonecrosis are loaded with VEGF. Secondly, an additional hastening of the already hurried revascularization and remodeling of the necrotic femoral head would speed up the structural and mechanical deterioration of the hip joint. On the contrary, it is mandatory to slow down the repair process as far as is feasible in order to conserve the greatest amounts of innate and biomechanically sufficient (albeit necrotic) epiphyseal bone for as long as possible, because accelerated bone turnover causes production of a mechanically frail osseous framework. Bone turnover should, therefore, be halted by medication with inhibitor(s) of VEGF, the prime intermediate in recruiting endothelial cell progenitors [102].

### 8. Medication with Zoledronic Acid

Little et al. have carried out a proficient series of experiments on the medication of rats with zoledronic acid (ZA) after surgically inducing osteonecrosis of the femoral head. They hypothesize that this bisphosphonate may preserve the structure of the femoral head while, at the same time, allowing incremental bone repair. Indeed, treatment and prophylaxis with ZA improve the sphericity and maintain the architecture of the necrotic femoral head. They have studied rats medicated subcutaneously with saline, ZA at one and 4 weeks after the operation (ZA-post), and ZA at 2 weeks pre-operation and at 1 and 4 weeks post-operation (ZA-pre-post). Six weeks postoperatively, 71% of the femoral heads of the saline-treated rats were aspherical. This contrasts with 13% and 0% aspherical femoral heads 6 weeks postoperatively in the ZA-post and ZA-pre-post animals (p < 0.05). Histomorphometrically, the bone volume was decreased by 12% in the saline group and close to 20% in the ZA-post and ZA-pre-post groups (p < 0.05). The retention of necrotic bone in the epiphyses of the treated rats was unambiguous. The difference between the non-treated and treated rats is explicitly due to the reduction in bone turnover [107].

### 9. Post-osteonecrotic Osteoarthritis-Like Disorder

Hip osteoarthritis is the leading treatment failure in children with Perthes disease and in adults with osteonecrosis. It results from the abnormal load transfer from the acetabulum to the femur across a remodeled and deformed femoral head. Contrary to clinicians' precepts, therapy that minimizes or hinders the remodeling processes delays the progressive deterioration of the articular structures. A balance between osteolysis and osteogenesis in the appropriate ratio is decisive in forestalling the collapse of the epiphysis, as preservation of the hemispherical shape of the femoral head is crucial in averting the development of osteoarthritis [61,62].

### 10. Concluding remarks

The means of treating osteonecrosis of the femoral head appraised in this review have been under experimental and clinical analyses for a few decades. Each of them has been praised at one time or another for providing the solution to the orthopaedic surgeons' frustrating deadlock in respect of restoring the necrotic femoral head to its earlier physical condition. In the rat model of vessel deprivation-induced osteonecrosis of the femoral head, medication with enoxaparin, construction of an intraosseous conduit, exposure to HBO and exposure to HBO plus NWB have been shown to hasten those reparative activities that are conventionally accepted as the epitome of revitalization of avascular dead bone. Investigators have a priori endeavored to enable vascular ingrowth. Accepting that osteonecrosis is caused by lack of blood supply, it is reasoned that the sooner the vasculature is reinstituted and delivery of oxygen and nutrients is returned to normal, the faster and more comprehensive would be the reconstruction of a living and mechanically well-performing femoral head. In mild cases, the femoral heads more or less retain their hemispherical profile. In more advanced cases, they are somewhat flattened or otherwise deviate from the hemispherical shape. Lastly, in the most severe cases, the femoral heads acquire any of a number of bizarre geometric forms. All repair processes are accelerated in rats treated by the above-mentioned means, including amassing undifferentiated mesenchymal cells, and profuse fibrogenesis, vasculogenesis, chondrogenesis and osteogenesis. At first sight it appears that all the clinically desired goals are attained. Alas, the profile of many a treated rat femoral head is disfigured to such a degree that smooth functioning of the hip joint is out of the question. The rising array of deformations correlates with an increasing extent of repair, indicating an inverse relationship between the degree of reconstruction and extent of revascularization on the one hand, and the magnitude of distortion of the femoral heads on the other. This explains the dictum that rats with maximally reconditioned necrotic femoral heads have the worst of it [103].

Brown et al. have given an account of the biomechanical properties of cancellous bone samples obtained from middle-stage and late-stage osteonecrosis of adult necrotic femoral heads. Compared to specimens retrieved from femoral heads of healthy individuals, samples removed from infarcted zones exhibit low yield strength, a much reduced elastic modulus and a modestly increased strain-to-failure. It is noteworthy that minor deviations in the strength and stiffness of bone taken from the affected regions are associated with large differences in the pattern of collapse and revascularization of the femoral heads [30]. An orthopaedic surgeon's dilemma is in which way to sway the modification of the remodeling necrotic bone without the usually-occurring decline in biomechanical properties, so that the structural distortion of the femoral heads is kept to a minimum.

The clinical relevance of animal experiments utilizing hyperoxygenation as the exclusive mode of treatment may be criticized because HBO in the clinical setting constitutes an adjunct to other therapeutic modalities. Incidentally, the outcome of studies of exposure of spontaneously hypertensive rats to HBO is irrelevant to our subject matter, because hyperoxygenation is utilized prophylactically [73]. As a rule, treatment in clinical practice commences after the symptoms and signs are overt, i.e. at a point in time when osteonecrosis is already comparatively advanced. In the studies cited herein, exposure to HBO was begun late in the course of the disease when the signs of osteonecrosis were already well developed.

Rats with vessel-deprived osteonecrosis of the femoral heads do not gain markedly from a NWB regimen. This concurs with the almost predictable collapse of the necrotic femoral heads in patients managed by restricted weight bearing [107]. While NWB by itself does not avert deformation of the femoral heads, the institution of HBO therapy in non-weight bearing rats often brings about a favorable outcome after 22 sessions of exposure to HBO. High oxygen tension is essential for osteogenesis to take place. Based on the documentation of raised osteolysis in mouse calvariae and rabbit femoral heads exposed to HBO, there is concern as to the biomechanical strength of the femoral heads after healing expedited by excess O_2_, in so far as too much osteolysis in too short a time may result in an untimely collapse of the femoral head [57,84,108,109]. Be this as it may, the deformation of the dead femoral heads in rats under weight bearing and exposure to HBO is less than that under NWB alone. These results are reminiscent of the enhanced mineralization and greater breaking strength of fractured femora in rats exposed to HBO and the greater mineral density of the bones and torsional strength of the tibiae of HBO-treated rabbits subjected to distraction osteogenesis [110,111].

The experimental mimickers of osteonecrosis of patient femoral heads possess certain distinctive traits, which differ from the disease as witnessed at the bedside. Osteonecrosis, with only a few exceptions, affects only part of the femoral head, while the epiphysis of rats virtually always undergoes total necrosis. Also, glucocorticoid-induced osteonecrosis in patients does not duplicate the coagulation-type death of the vascular deprivation-induced disorder in rats, but rather evinces an apoptotic process [112]. However, Glueck and coworkers have indicated that thrombophilia may cause thrombotic occlusion of veins, accompanied by venous hypertension of the marrow and hypoxic bone death, so that Perthes disease ensues [113].

## The Role of Glucocorticoids, Rank-Ligand and Apoptosis

It is well known that glucocorticoids suppress osteogenesis and cause calcium loss. Yet the mechanism(s) linking glucocorticoid medication to osteonecrosis of the femoral head remain mystifying. It is fascinating that idiopathic and glucocorticoid-induced osteonecrosis cannot be distinguished histologically, maybe because at least some intermediate events are common to both [114]. Fat embolization may lead to osteonecrosis in patients with lipid disorders as well as those with glucocorticoid-induced hyperlipidemias [115]. Jones has described the triad of intraosseous fat embolism, intravascular coagulation and osteonecrosis in man. He has suggested that the mediatory pathway of fat emboli, hypercoagulability, stasis and endothelial damage by free fatty acids induces osteonecrosis by gradually occluding subchondral capillaries and sinusoids with fibrin-platelet thrombi [116]. Drescher and coworkers have proposed that the declining epiphyseal blood flow and the raised plasma fibrinogen after an infusion of high doses of methylprednisolone is causal in the early stage of steroid-induced osteonecrosis in the conscious pig [117].

The ratio of cytokines promoting the differentiation of osteoclasts to those blocking this process is raised in glucocorticoid-treated osteoblasts. These cytokines include the TNF-α family, RANKL and osteoprotegerin [115,118]. It is yet undecided how they are linked to the site-specific damage caused by glucocorticoids. Osteoblasts and osteocytes of the femoral cortex in mice, and the iliac bones of patients prescribed glucocorticoids for some years, undergo apoptosis [119]. Nevertheless, isolated osteoblasts behave erratically in that they often do not display the expected glucocorticoid-induced apoptosis. In fact, glucocorticoids have been employed to stimulate precursor cells to differentiate into osteoblasts [120].

It is challenging to determine how glucocorticoids bring about widespread osteolysis on the one hand, and cause damage to distinctive skeletal sites – say, the femoral head – on the other. Experimental data imply that glucocorticoid-induced apoptosis of osteocytes coincides with vascular blockage-independent osteonecrosis [121]. By itself, a positive TUNEL reaction does not discriminate among the manifold causes of cell death. It is often associated with a synchronized pattern of clustered dead osteocytes and changes in the osseous matrix. The hyperpermeability of the matrix (confirmed by the lamellar adsorption of tetracycline) recalls the fact that osteocytes before and throughout the apoptotic process participate in the degradation of the environment. Osteocytes produce collagenases in vitro, but their capacity to release proteolytic enzymes in vivo is as yet unknown [122].

Glucocorticoid-promoted expression of the RANK-ligand by the osteoblasts represents a central pathway in osteoclast maturation [123]. The activation of the RANK-ligand is linked to the injured bony matrix, increased permeability of the matrix, and fragmentation of osteoblast and osteocyte DNA. The differentiation factors accelerating the osteoclastic activities seem to originate from the residual living stromal cells surrounding the apoptotic bone cells. The stromal cells of the marrow produce RANK-ligand and other key osteoclast-modifying cytokines, including those that stimulate bone turnover [124]. The in vivo survival and differentiation of osteoblasts depend on high glucocorticoid levels. Long-term corticosteroid medication-induced apoptosis occurs in murine femoral cortex and patients' iliac bones. Fewer than 1% of the trabeculae-lining osteoblasts are TUNEL-positive in healthy bone. While the cortex is normal in methylprednisolone-treated rats, the trabecular bone is undergoing resorption and a large fraction of its osteoblasts and osteocytes are TUNEL-positive. It should not go unnoticed that the TUNEL technique has an important shortcoming in so far as it indicates DNA fragmentation, which is not only a late occurrence in the apoptotic cascade but might also reflect other mechanisms of cell death [119,125].

Glucocorticoids play a prominent physiological role in the turnover of healthy bone. The process of osteoblastic maturation in tissue cultures is stimulated by glucocorticoids at the proper concentration [122,126]. In view of experimental data from rabbits, Eberhardt et al. have reasoned that apoptosis of the bone cells necessarily entails excessive stimulation of a crucial receptor [127].

Trabecular bone bends under a load to a greater extent than cortical bone. Hence, the especial sensitivity of trabecular osteoblasts and osteocytes possibly reflects variations in the microenvironment. Lastly, proapoptotic signals such as NO_2_, generated by mechanically strained osteoblasts and osteocytes, are likely to affect the response to glucocorticoids [128]. Based on their experimental data, Weinstein and his colleagues hypothesize that bone in the subarticular trabeculae of the femoral head is especially sensitive to glucocorticoid-induced apoptosis because it has a large active surface area under constant high stress load [119].
